# Comorbid Anxiety Increases Suicidal Risk in Bipolar Depression: Analysis of 9720 Adolescent Inpatients

**DOI:** 10.3390/bs10070108

**Published:** 2020-07-04

**Authors:** Ozge Ceren Amuk, Rikinkumar S. Patel

**Affiliations:** 1 Department of Psychiatry, School of Medicine, Koç University, Davutpaşa Caddesi No. 4 Topkapı, İstanbul 34010, Turkey; drozgeceren@gmail.com; 2 Department of Psychiatry, Griffin Memorial Hospital, Norman, OK 73071, USA

**Keywords:** suicidality, adolescents, bipolar depression, bipolar disorder, depressive episode

## Abstract

Objective: To evaluate the risk of association between suicidal behaviors and comorbid anxiety disorders in adolescents with bipolar depression. Methods: We conducted a cross-sectional study using the nationwide inpatient sample (NIS) from the United States. This study included 9720 adolescent inpatients with bipolar depression and further grouped by co-diagnosis of anxiety disorders. Logistic regression analysis was used to evaluate the odds ratio (OR) of suicidal behaviors due to comorbid anxiety after controlling demographic confounders and psychiatric comorbidities. Results: Out of total inpatients, 34.8% (*n* = 3385) had comorbid anxiety disorders with a predominance in females (70.3%) and White patients (67.7%). About 54.1% of inpatients with comorbid anxiety had suicidal behaviors versus 44.6% in the non-anxiety cohort (*p* < 0.001). Comorbid anxiety disorders were associated with 1.35 times higher odds (95% CI 1.23–1.47, *p* < 0.001) for suicidal behaviors. Conclusion: Suicidal behaviors are significantly prevalent in bipolar depression adolescents with comorbid anxiety disorders. Anxiety disorders are an independent risk factor in bipolar depression that increase the risk of suicidal behaviors by 35%. This necessitates careful assessment and management of comorbid anxiety disorders in bipolar youth to mitigate suicidality.

## 1. Introduction

Bipolar disorder (BP) is a psychiatric condition that causes irregular changes in mood, motivation, levels of activity, attention, and the ability to perform everyday tasks [1]. BP is a recurring familial disorder that occurs in 1–3% of adolescents. It is difficult to diagnose BP in young people due to the immaturity of the individual, the existence of comorbid conditions, and divergent definitions of manic symptomatology [2]. Most adolescents with BP experienced a depressive episode as the onset mood phase with the mean age of onset at 11.75 years. The incidence of BP rose with age, with an almost double rise from the age group 13 to 14 to those between 17 to 18 years [3].

Suicidal behaviors are high among patients with BP as about 25% to 50% adults with BP had at least one lifetime suicide attempt and eight to 19% of died from suicide [4]. About 20% to 65% of adults with an initial BP experience in childhood and those with an early onset of disease are at greater risk for suicidal behaviors [4]. The prevalence of current and past suicidal ideation in pediatric BP are 50% and 57%, respectively, and are significant risk factors for a suicidal attempt [5]. The worsening of BP symptoms and poorer functioning, and comorbidities including anxiety, attention deficit hyperactivity disorder (ADHD) and substance use disorders (SUD) were associated with a suicidal attempt [6]. Patients with BP faced double the risk of suicide relative to individuals with other depression types [7]. Adolescents with BP had a weighted mean average of current suicide ideation of 50.4% and 25.5% for suicidal attempts [8]. Despite the correlation between early onset of BP and suicidality, little is known about underlying pathophysiology for higher suicide risk in bipolar depression adolescents [4].

In the systematic treatment enhancement program for bipolar disorder (STEP-BD), the lifetime prevalence for comorbid anxiety disorders in adult patients with BP was 51.2% [9]. A systematic review reported that comorbid anxiety is prevalent in of 41% to 80% of pediatric BP. In addition, family studies have confirmed higher levels of anxiety disorders in children of parents with BP [10]. Adolescents with BP and comorbid anxiety have significant mood symptoms and lower recovery levels relative to adolescents with no comorbid anxiety [11]. BP and comorbid anxiety are correlated with worse patient outcomes, including higher levels of rapid cycling, more severe depressive episodes, risk of SUD and suicide attempts, as well as lower rates of response to treatment [11,12]. Biological variables, cognitive growth, family and peer relationships, and self-perception can influence symptoms of anxiety. As adolescent development involves many features that may be specific to understanding anxiety disorders, studying these interactions can be crucial to determine the etiology of lifelong anxiety disorders [13].

The exact nature of the relation between anxiety and BP, nevertheless, remains uncertain. Anxiety can be a prodromal symptom of BP, or alternatively anxiety and BP can share a biological or genetic risk [9]. The precise mechanism by which anxiety elevates suicidality is little understood. Patients with acute anxiety may be less likely to handle negative consequences, and use other tools to minimize suicidality, such as psychological services or cognitive techniques [9]. A demonstration of link between comorbid anxiety with higher severity and impairment in patients with BP shows the need for greater clinical attention to anxiety in this population, especially for increased clinical suicidal monitoring [9].

Our study goals are to (1). understand the demographic distribution and psychiatric comorbidities in adolescents with bipolar depression and comorbid anxiety disorders, and (2). To evaluate the risk of association between suicidal behaviors and comorbid anxiety disorders in adolescents with bipolar depression.

## 2. Methods

### 2.1. Data Source

We conducted a cross-sectional analysis using the national inpatient sample (NIS) from January 2012 to December 2014 [14]. The NIS data includes inpatient data from 4400 non-federal hospitals across 45 states in the US. The primary and co-diagnostic information is identified using the international classification of diseases, ninth revision (ICD-9) and clinical classification software (CCS) codes [14]. The NIS is a de-identified dataset and so this study does not require approval from the institutional review board [14].

### 2.2. Inclusion Criteria and Outcome Variables

We included 9720 adolescent inpatients (age 12 to 17 years) with a primary diagnosis of bipolar depression using the ICD-9 codes: 296.50–296.56. The study population was further sub grouped based on co-diagnosis of anxiety disorders using the ICD-9 codes 293.84, 300.00–300.02, 300.09, 300.10, 300.20–300.23, 300.29, 300.3, 300.5, 300.89, 300.9, 308.0–308.4, 308.9, 309.81, 313.0, 313.1, 313.21, 313.22, 313.3, 313.82 or 313.83. The co-diagnosis of suicidal behaviors was detected in the patient records using ICD-9 codes for suicidal ideation (V62.84) and suicide and self-inflicted injury (E950.XX–E959.XX) [15].

We included demographic characteristics: age, sex (male or female), and race (White, Black, Hispanic, and Native American (NA)/Asian [16]. The comorbidities studied are attention-deficit/hyperactivity disorder (ADHD)/conduct/behavioral disorders (CCS code: 652), psychotic disorders (CCS code: 659), alcohol abuse (CCS code: 660) and substance abuse (CCS code: 661) [16].

### 2.3. Statistical Analysis

We used descriptive statistics and Pearson’s chi square test to measure the differences in demographic and psychiatric comorbidities, and suicidal behavior in bipolar depression inpatients by presence of comorbid anxiety disorders. Logistic regression analysis was used to evaluate the odds ratio (OR) of suicidal behaviors in inpatients with versus without comorbid anxiety disorders after controlling demographic confounders and other psychiatric comorbidities. A *p*-value < 0.01 was the standpoint for statistical significance in all the analyses that was done on SPSS version 26 (IBM Corporation, Armonk, NY, USA).

## 3. Results

Our study population of 9720 bipolar depression inpatients was mainly female (62.1%) and White (64.8%). Out of the total inpatients, 34.8% (*n* = 3385) had comorbid anxiety disorders. A higher proportion of these inpatients with comorbid anxiety disorders were females (70.3%), compared to 57.8% females without comorbid anxiety. Additionally, bipolar depression inpatients with comorbid anxiety was most prevalent in White patients (67.7%) followed by Hispanic (16.3%) and Black patients (11.2%), and there was statistically no significant difference when compared with the non-anxiety cohort.

About 54.1% bipolar depression inpatients with anxiety had suicidal behaviors which was significantly higher than that seen in the 44.6% of the non-anxiety cohort (*p* < 0.001). The most prevalent psychiatric comorbidities in the anxiety cohort was ADHD/conduct disorder/disruptive behavioral disorders (41.5%), but there was not a statistically significant difference when compared with the non-anxiety cohort (*p* = 0.370). Substance abuse was seen in a significantly higher proportion of inpatients with anxiety (18.3% vs. 15.5% in non-anxiety, *p* < 0.001). There was statistically no significant difference in the prevalence of comorbid psychotic disorders and alcohol abuse, as shown in Table 1.

Predictors of suicidal behaviors in bipolar depression

1. Age: It had no significant association with suicidal behaviors (*p* = 0.212).
2. Sex: Females had 1.38 times higher odds compared to males (95% CI 1.27–1.51, *p* < 0.001).
3. Race: Hispanic patients had 1.52 times (95% CI 1.33–1.73, *p* < 0.001) and NA/Asian patients had 1.36 times (95% CI 1.14–1.63, *p* = 0.001) higher odds compared to White patients.
4. Comorbidities: Psychotic disorder was associated with 1.59 times higher odds (95% CI 1.26–2.02, *p* < 0.001) for suicidal behaviors. Alcohol abuse and substance abuse relationships with suicidal behaviors was not statistically significant.
5. Anxiety: Comorbid anxiety disorders were associated with 1.35 times higher odds (95% CI 1.23–1.47, *p* < 0.001) for suicidal behaviors after controlling for demographic confounders and psychiatric comorbidities, as shown in Table 2.

## 4. Discussion

Demographics and psychiatric comorbidities play an important role in adolescents with bipolar depression having suicidal behaviors. We analyzed the largest inpatient data from the US hospitals and found that females (increased by 38%) and Hispanic (increased by 52%) with bipolar depression have higher risk for suicidal behaviors. Comorbid anxiety disorder acts an independent risk factor for increasing the likelihood for suicidal behaviors by 35%.

By gender distribution, 62.1% patients with bipolar depression were females, with a male–female ratio of 2:3 as per our data analysis. This correlates with past studies that found the prevalence of BP was higher in females [17]. Biological changes from preadolescence to adulthood may affect the appearance of anxiety disorders in adolescence. For instance, variations in anxiety symptomatology between adolescent males and females were postulated to be linked to advancing sexual growth and associated changes in sex hormones [18]. During the early stages of puberty, anxiety symptoms frequently increase and continue into young adulthood [18].

As per a study in adolescent outpatient mental health service, BP was more prevalent in White patients, as adolescents from minority groups may have limited access to mental health services with a lower diagnosis rate [19]. Additionally, there exists gaps in parental awareness of child mental health issues, medical care needs, treatment costs and insurance coverage which leads to inequalities in the use of mental health services [19]. Furthermore, in our study of inpatients, about 65% of patients were White, though there was not a statistically significant difference across race for the co-diagnosis of anxiety disorders in bipolar depression.

The lifetime prevalence of comorbid anxiety disorders in adolescents with BP was between 14% and 56% [11]. In our study, we only included bipolar depression adolescents and found 34.8% of adolescents with comorbid anxiety disorders, which closely relates with a past study by Sala et al. [11]. A higher proportion of bipolar depression inpatients with anxiety were females (70.3%) and White patients (67.7%). Seminal results from a female twin registry indicate that genetic factors pose a major threat for women’s anxiety disorders [20]. Additionally, early-life trauma, such as sexual or physical violence during adolescence, predisposes anxiety disorders to develop later in life [21]. Our results do not address the impact of the severity of differential anxiety, or the characteristics of subsyndromal anxiety. Symptoms of anxiety below the threshold to meet medical criteria may also have a detrimental impact on the path of bipolar disorder [9]. For instance, lifelong panic spectrum symptoms and features, independent of formal diagnosis of panic disorder, are associated with higher bipolar severity and longer time to recovery [9]. Further research to address effect of subgroups of anxiety disorders and its severity on comorbid bipolar disorder is needed.

Individuals from minorities, especially Hispanic and Asian groups, are less likely to meet the criteria for anxiety disorders than White Americans. White patients were more likely to be diagnosed with social anxiety disorder, generalized anxiety disorder, and panic disorder than Black, Hispanic, and Asian patients. Another significant factor declared by the World Health Organization’s Composite International Diagnostic Interview (WHO WMH-CIDI) is the fact that there is no sufficient analysis of understanding of anxiety symptoms in terms of linguistic or cultural variations in these populations [22]. The discrepancies found in this analysis between ethnic groups may also reflect underlying differences in the specific diagnostic criteria for the anxiety disorders being tested [22].

Studies in non-American populations have found prevalence levels for anxiety disorders to be significantly different from those seen in large-scale epidemiological studies in the US, which more readily suggest methodological errors, some form of response bias, or, as mentioned above, a disparity in the perceived experience of those disorders [23]. Another factor which may account for differences in prevalence levels for immigrant populations versus their counterparts in native settings is the degree of individualistic or collectivist identity of the assessed individuals [24,25]. There exists differences in the rate of anxiety in societies that identify with more collectivist values, where the emphasis on preserving unity within the community is of the highest importance compared to those who adhere to more individualistic cultural attitudes, where individual accomplishment is highly valued and rewarded by the rest of the social group [24,25]. An evaluation of the degree of commitment to primarily individualistic or collectivistic principles may thus have an effect on the recording of symptoms of anxiety.

Anxiety disorders and substance abuse commonly co-occur with at least one illicit substance abused in 15% of those with anxiety disorders [26]. In our study, substance abuse was seen in a significantly higher proportion of adolescents with bipolar depression and anxiety (18.3% vs. 15.5% in non-anxiety). In previous studies, it was found that young individuals with anxiety disorders are at a higher risk of substance abuse, and this association is non-causal and contributed due to risk factors such as child neglect, family trauma exposure, and parental psychopathology, as well as previous substance abuse, self-medication, and peer burden [27].

The prevalence of suicidal behaviors in adolescents with BP was 50.4% for suicidal ideations and 25.5% for suicidal attempt [8], and this correlates with our findings of a prevalence of suicidal behaviors seen in 54% of adolescents with bipolar depression and comorbid anxiety disorders. Comorbid anxiety increases the risk of suicidal behaviors in bipolar depression adolescents by 46% and, after the regression model was adjusted for demographic confounders and psychiatric comorbidities, the risk of association with suicidal behaviors was persistently statistically significant and increased by 35%. BP patients in mixed states with suicidal ideation was seen in 62.8% of females, 69.4% in those with a past history of a suicide attempt [28]. Females have more than double the risk of suicidal ideation, and almost three times the risk of a history of a suicide attempt compared to males in an adult BP study [28]. In our study, females with bipolar depression had 1.4 times higher odds for suicidal behaviors compared to males. The rapid elevation in acute anxiety-related symptoms, like elevated arousal/agitation and panic symptoms, can dramatically increase the likelihood of suicidality [28]. In contrast, some adult studies showed an association between past suicide attempts and comorbid anxiety in bipolar disorder [29,30,31,32,33,34]. One adult study found no evidence that a comorbid anxiety disorder was associated with greater likelihood of a past suicide attempt or more severe suicidal ideation in bipolar disorder [35]. Additionally, it is also argued that comorbid anxiety has a protective effect on suicidal behavior in a mixed major depressive disorder (MDD) and bipolar sample and in a pure MDD sample [36,37]. The discrepancies between the results of different studies may be due to variations in sampling, which may affect anxiety-related effects. Furthermore, although there are a number of clinical rating scales for the severity of suicidality, only a few have been validated in children and adolescents [38].

Anxiety and related symptoms over a shorter period could be better predictors of suicidal thoughts and behaviors [28]. Comorbid anxiety disorders and childhood maltreatment have worse outcomes of BP with increased severity and suicidal attempts [39]. The disease burden in adolescents with BP and comorbid anxiety is higher than those with major depressive disorder and comorbid anxiety. The number of comorbidities, including anxiety disorders, suicidality, psychosis and substance use, was strongly correlated with the severity of the mood disorders [40]. Comorbid anxiety disorders increase the probability of a relapse of a major depressive episode or a manic episode, and suicidal ideation due to the worsening severity of the disease with reduced functioning, reduced remission rate, and sleep disturbances [41]. Our study has an importance of analyzing comorbid anxiety in particularly pediatric population with bipolar depression. Thus, further studies needed to assess in pediatric population and follow up patients in longer terms to analyze this correlation better.

The National Comorbidity Survey for lifetime suicide attempts showed that Hispanic Americans had a higher risk of suicidal thoughts, intentions, and impulsive attempts relative to White Americans, though the frequency was not higher for suicide attempts [42]. This is well-supported by our study, as Hispanic patients had 1.5 times, and Asian and/or Native American patients had 1.4 times higher odds compared to White ethnicities for suicidal behaviors. Additionally, as per a study in the US, Hispanic and Asian Americans are less likely to receive mental health care than White Americans [43].

Pediatric BP with psychotic symptoms are associated with suicidal attempts along with a greater severity of the disease and psychiatric hospitalizations [5]. About 93.2% adolescents with suicidal attempt had bipolar depression, whereas only 6.8% had unipolar depression and so accurate diagnosis of depression type is important for management [44]. In our study, bipolar depression adolescents with comorbid psychotic disorders were at 1.6 times higher odds for suicidal behaviors. The correlation between suicidal behaviors and substance use is seen in both adolescent and adult studies due to the immaturity of effective, cognitive, and behavioral processes in adolescents. Impulsive and risk-taking behaviors worsen with higher substance use, increasing the risk of suicidal behaviors [45]. However, in our study, there was a very small positive association between suicidal behavior with comorbid alcohol and substance abuse, but the was not statistically significant after controlling with demographic confounders and other psychiatric comorbidities.

Our study has a many limitations. Firstly, we could not quantify the course of illness, previous depressive episodes, and duration between bipolar depression and emergence of anxiety symptoms. Secondly, we did not differentiate the suicidal behaviors between suicidal ideation versus attempts. Thirdly, we have only included bipolar depression based on ICD-9 diagnostic codes, and excluded BP, manic episodes pertaining to our study objectives. The international classification of diseases, 11th revision (ICD-11) differentiates hypomania from mania on the basis of seven days duration but, sets the lower limit for hypomania at “several” days [46]. Since patients with BP type II have a lower rate of switching on antidepressants than those with BP type I, distinguishing between these disorders is important from a pharmacological aspect [47].

One of the major limitations includes the possibility of over-reporting of bipolar depression and comorbid anxiety disorders, as patients with BP may have extremes of deep depression and normality versus extreme excitement and overactivity. On the other hand, an irrational excitement and optimism of the manic phase with anxiety neurosis would exclude the presentation of classic symptoms of anxiety leading to under-reporting of comorbid anxiety disorders. In our cross-sectional study, there may be a possibility of the diagnoses of bipolar depression be based on episodes of severe and agitated depression with high levels of anxiety, and desire for self-harm.

Additionally, subclasses of anxiety disorders were not specified in our study. After the DSM-5, there are phenomenological differences between disruptive mood dysregulation disorder (DMDD) and pediatric BP, and the exclusion of obsessive-compulsive disorder from the anxiety disorders category also limit the generalization of the ‘any anxiety disorder’ group [2]. Nevertheless, clinical trials remain relevant, as incredibly large population studies will be required to research some of the interesting comorbidities due to the relative rareness of certain anxiety disorders. Further, such retrospective studies are always subject to selection bias, which might be accentuated by the moderate sensitivity of diagnostic codes for bipolar depression [48]. However, the biggest strength of our study lies in the national representation of the dataset, with a uniform collection of data through ICD-9 codes. This is the first study, to our knowledge, to report the impact of comorbidities and demographics on the link between comorbid anxiety disorders and determining suicidal behaviors in bipolar depression adolescents.

## 5. Conclusions

Considering prevalence and burden of bipolar depression with comorbid anxiety disorders in the adolescent population, the importance of determining demographic predictors and comorbidities of the disease is increasing. Suicidal behaviors are significantly prevalent in bipolar depression adolescents with comorbid anxiety disorders. Anxiety disorders are an independent risk factor in bipolar depression that increases the risk of suicidal behaviors by 35%. This necessitates careful assessment and management of comorbid anxiety disorders in bipolar youth to mitigate suicidality.

## Figures and Tables

**Table 1:** Demographic and comorbidities in bipolar depression inpatients.

Variable	Comorbid Anxiety Disorders			*p* Value
	(−)	(+)	Total	
Total N	6335	3385	9720	-
Mean age, years	14.85	14.90	-	0.218
Sex, %				
Male	42.2	29.7	37.9	<0.001
Female	57.8	70.3	62.1	
Race, %				
White	63.2	67.7	64.8	0.183
Black	19.1	11.2	16.4	
Hispanic	11.2	16.3	13.0	
NA/Asian	6.5	4.8	5.9	
Comorbidities				
ADHD/conduct/behavioral disorder	40.6	41.5	40.9	0.370
Psychotic disorders	3.4	3.4	3.4	0.993
Suicidal behaviors	44.6	54.1	47.9	<0.001
Alcohol abuse	5.4	5.5	5.4	0.838
Substance abuse	15.5	18.3	16.5	<0.001

The proportion in bipolar depression inpatients were obtained using cross tabulation and the Pearson Chi-Square (χ^2^) test with significant *p* values ≤ 0.01. NA: Native American; ADHD: attention deficit hyperactivity disorder.

**Table 2:** Predictors of suicidal behaviors in bipolar depression inpatients.

Variable	Logistic Regression Analysis		
	OR	95% CI	*p* Value
Age	1.0	0.99–1.04	0.212
Sex			
Male	Reference		
Female	1.38	1.27–1.51	<0.001
Race			
White	Reference		
Black	0.79	0.70–0.89	<0.001
Hispanic	1.52	1.33–1.73	<0.001
NA/Asian	1.36	1.14–1.63	0.001
Comorbid anxiety disorder			
No	Reference		
Yes	1.35	1.23–1.47	<0.001
Comorbidities			
ADHD/conduct/behavioral disorder	0.98	0.91–1.07	0.668
Psychotic disorders	1.59	1.26–2.02	<0.001
Alcohol abuse	1.08	0.88–1.33	0.459
Substance abuse	1.03	0.91–1.17	0.644

Odds ratio was generated by logistic regression model and was adjusted for age, gender, race, and psychiatric comorbidities. Significant *p* values ≤ 0.01 at 95% Confidence Interval. NA: Native American; ADHD: attention deficit hyperactivity disorder; OR: odds ratio; CI: confidence interval.
